# Monitoring innate immune cell dynamics in the glioma microenvironment by magnetic resonance imaging and multiphoton microscopy (MR-MPM)

**DOI:** 10.7150/thno.38659

**Published:** 2020-01-01

**Authors:** Kianush Karimian-Jazi, Philipp Münch, Allen Alexander, Manuel Fischer, Kira Pfleiderer, Manuel Piechutta, Matthia A. Karreman, Gergely M. Solecki, Anna S. Berghoff, Mirco Friedrich, Katrin Deumelandt, Felix T. Kurz, Wolfgang Wick, Sabine Heiland, Martin Bendszus, Frank Winkler, Michael Platten, Michael O. Breckwoldt

**Affiliations:** 1 Neuroradiology Department, University Hospital Heidelberg, 69120 Heidelberg, Germany.; 2 Clinical Cooperation Unit Neurooncology, German Cancer Consortium (DKTK) within the German Cancer Research Center (DKFZ), 69120 Heidelberg, Germany.; 3 Clinical Cooperation Unit Neuroimmunology and Brain Tumor Immunology, German Cancer Research Center (DKFZ), 69120 Heidelberg, Germany.; 4 Neurology Clinic and National Center for Tumor Diseases, University Hospital Heidelberg, 69120 Heidelberg, Germany.; 5 Division of Oncology, Department of Medicine I, Medical University of Vienna.; 6 Department of Neurology, University Medical Center Mannheim, Heidelberg University, Theodor-Kutzer-Ufer 1-3, Mannheim, Germany.

**Keywords:** MRI, multiphoton microscopy, tumor microenvironment, tumor-associated macrophages, iron oxide nanoparticles, immunotherapy.

## Abstract

**Rationale:** Glioblastoma is the most frequent, primary brain tumor that is characterized by a highly immunosuppressive tumor microenvironment (TME). The TME plays a key role for tumor biology and the effectiveness of immunotherapies. Composition of the TME correlates with overall survival and governs therapy response. Non invasive assessment of the TME has been notoriously difficult. **Methods:** We have designed an *in vivo* imaging approach to non invasively visualize innate immune cell dynamics in the TME in a mouse glioma model by correlated MRI and multiphoton microscopy (MR-MPM) using a bimodal, fluorescently labeled iron oxide nanoparticle (NP). The introduction of Teflon cranial windows instead of conventional Titanium rings dramatically reduced susceptibility artifacts on MRI and allowed longitudinal MR-MPM imaging for innate immune cell tracking in the same animal. **Results:** We visualized tumor associated macrophage and microglia (TAM) dynamics in the TME and dissect the single steps of NP uptake by blood-born monocytes that give rise to tumor-associated macrophages. Next to peripheral NP-loading, we identified a second route of direct nanoparticle uptake via the disrupted blood-brain barrier to directly label tissue resident TAMs. **Conclusion:** Our approach allows innate immune cell tracking by MRI and multiphoton microscopy in the same animal to longitudinally investigate innate immune cell dynamics in the TME.

## Introduction

Glioblastoma is the most malignant, primary brain tumor entity and is characterized by an immunosuppressive tumor microenvironment (TME) 1, 2. The TME is composed of various stroma and immune cells that interact heavily within the tumor. Part of these non-neoplastic constituents of the immune system are tumor-associated macrophages and microglia (TAMs) 3, 4. TAMs are the dominant infiltrating immune cell population in glioblastoma and show multiple reciprocal interactions with tumor cells to promote tumor growth and progression 5. The composition of the TME and the amount of *e.g.* cytotoxic CD8 T cells or regulatory T cells has been identified as a predictive marker for survival and therapy response in various solid cancers including glioma. Thus, the TME is a promising target for therapeutic interventions, such as immune modulating therapies 6-8. Gliomas are characterized by an immunosuppressive microenvironment that show large infiltrates of M2-like macrophages / microglia 1, 2, 9. Recently, a number of novel immunotherapies have been developed for glioma that modulate the tumor environment and exploit various immunotherapeutic strategies mainly targeting the adaptive immune system [3, 4, 10). Macrophages and microglia serve as antigen presenting cells and phagocytose tumor debris 1, 5. Innate immune cells are actively modulated by the tumor towards an anti-inflammatory (“M2-like”) phenotype, thus mediating tumor immune escape. Additionally, M2-like macrophages can produce a plethora of cytokines and chemokines that can further stimulate tumor growth, *e.g.* by the secretion of pro-angiogenic factors like vascular endothelial growth factor (VEGF). Monitoring anti-tumor immune responses is a major challenge in clinical practice 6-8, 11. Imaging is the main modality to monitor solid tumors but functional methods to monitor cellular and molecular changes in the TME have been limited so far 12. Iron oxide nanoparticles that can be detected by MRI have been shown to accumulate in phagocyte subsets and therefore allow monitoring of immune responses 13. We have previously established iron oxide nanoparticle (NP) imaging in a model of multiple sclerosis using dextran coated, cross-linked iron oxide NPs conjugated with fluorescent dyes as a bimodal sensor of innate immune cells 14. We combine this strategy with *in vivo* multiphoton microscopy (MPM) through a chronic cranial window 15. MPM has been widely used for *e.g.* deep tissue imaging, mapping of neuronal activity and studying cellular interactions down to the subcellular level 16-19. Using this dual-imaging approach we visualize the cellular and subcellular dynamics of nanoparticle uptake and sequestration. To achieve this goal of dual modality imaging by MRI and MPM (MR-MPM) we developed a new cranial window technique for MPM to reduce metal artifacts in MRI. Mandatory MPM head holders built from Titanium, a paramagnetic material that is also commonly used for human implants, result in prohibitive metal artifacts which are particularly strong in sequences that are used for visualizing iron oxide NP. We reasoned that Teflon rings, which are not paramagnetic, do not show susceptibility artifacts and thus allow correlated recordings of MRI and MPM. Using this approach we obtained high field MRI at 9.4 Tesla and *in vivo* multiphoton microscopy in the same animal to assess the TME from the macro- to the sub-µm scale. We show that NP signals are specific for the innate immune cell compartment and decipher various routes of NP uptake by circulating monocytes, tumor infiltrating macrophages and tumor microglia to yield an integrative view of innate immune cell dynamics in the glioma TME.

## Methods

### Cell culture

Gl261 cells were purchased from the National Cancer Institute Tumor. Gl261 cells were cultured in Dulbecco's modified Eagle's medium (DMEM) supplemented with 10 % fetal bovine serum (FBS) and 100 U/ml penicillin and 100 µg/ml streptomycin (all Sigma-Aldrich) at 37°C, 5% CO_2_. Gl261 cells were routinely tested for viral, mycoplasma and non-murine cell contamination by the multiplex cell contamination test (Multiplexion GmbH) 20.

### Glioma mouse model

We used 8-12 week old C57black6-J WT mice, purchased from Janvier Laboratories (Le Genest Saint Isle, France) or heterozygous Cx3cr1-GFP^+/-^ mice 21 with GFP-labeled myeloid cells (monocytes, macrophages and microglia). C57black6 mice were injected intracranially with 50.000 Gl261-GFP tumor cells in 2 µl PBS and red-fluorescent NP (cross-linked iron oxide nanoparticle labeled with 5-carboxytetramethylrhodamine, CLIO-TAMRA) to assess a possible NP uptake by glioma cells. Cx3cr1-GFP^+/-^ mice were engrafted with non-fluorescent Gl261 cells and intravenously injected with CLIO-TAMRA to assess NP uptake by macrophages/ microglia. Mice were monitored clinically and animals were sacrificed if they showed any signs of neurological symptoms or weight loss >20%. All efforts were made to minimize animal suffering and to reduce the number of animals used. All animal procedures were performed in accordance with the institutional laboratory animal research guidelines and with the approval of the governmental authorities (Regierungspräsidium Karlsruhe, Germany; approval number: G27-17).

### Cranial window and tumor implantation

For the preparation of the cranial window 22-24, mice were anesthetized with 100 mg/kg ketamine i.p. and 10 mg/kg xylazine i.p. Eyes were covered with an eye ointment (Bepanthen®) and body temperature was maintained by a heating pad. The skull was stably immobilized in a skull holder using metal ear sticks. All instruments and materials used were sterilized before each operation. The surgical steps were carried out under stereomicroscopic control. The scalp was disinfected with iodine-containing disinfectant solution and removed with scissors from the level of the eyes to the neck muscles. After using a cover glass as a template to plot the drilling marks the skull was drilled. The drill and skull bone was constantly cooled with saline solution. The bone lid was carefully lifted and rinsed off the dura mater. Minor bleedings from superficial cerebral veins were stopped using a hemostatic fleece (Tachosil®). The brain surface was constantly moistened with saline. The bone defect was closed and sealed with a coverslip using acrylic adhesive. The newly constructed Teflon ring was attached with acrylyc adhesive. The ring allowed painless and stable fixation while imaging. After the surgery mice received 2.5 mg/kg carprofen s.c., 8mg/kg moxifloxacin s.c. and 0.5mg/kg dexamethasone s.c. Operated mice were transferred to fresh cages and housed individually. The analgesic treatment with 5 mg/kg carprofen s.c. was continued twice daily for at least 48 hours. The intracranial tumor cell implantation was performed ~three weeks after surgery of the chronic cranial window when mice had fully recovered. To avoid metal artifacts on MR images we replaced the conventional Titanium ring with a custom-made polytetrafluorethylene (PTFE, Teflon^®^) ring. Additional rings were manufactured from polymethylmethacrylate (PMMA), polyacetale (POM), polyamide (PA) and polypropylene (PP) by the in-house mechanical workshop of the University clinic of Heidelberg. For *in vitro* testing of MR imaging properties rings were embedded in 3% agarose (Sigma) in 15ml falcon tubes and measured at 9.4T using T2 and T2* sequences. For *in vivo* experiments size, thickness and fixation of the ring were identical as for the standard titanium ring, including glue application and implantation of the window. 2-3 weeks after cranial window implantation, 50.000 Gl261 glioma cells were stereotactically injected into the frontal cortex at a depth of 500μm (coordinates 2mm lateral and 2mm ventral from bregma).

### MR imaging

MR imaging was performed on a 9.4 Tesla horizontal bore small animal NMR scanner (BioSpec 94/20 USR, Bruker BioSpin GmbH, Ettlingen, Germany) with a mouse brain array RF-coil (2x2 array). MR imaging included a standard rapid acquisition with relaxation enhancement (RARE) T2-w and T1-w post-Gadolinium (Gd)-contrast sequence to monitor tumor volume (T2-w parameters: 2D sequence, TE: 33 ms, TR: 2500 ms, flip angle: 90°, acquisition matrix: 200 x 150, number of averages: 2, slice thickness: 700 µm, acquisition time: 2 min 53 sec; T1-w parameters: TE: 6 ms, TR: 1000 ms, flip angle: 90°, acquisition matrix: 256 x 256, number of averages: 2, slice thickness: 500 µm, acquisition time: 5 min). We also used a T2-w 3D-RARE sequence: TE 33 ms; TR: 1,800 ms; flip angle: 90°; acquisition matrix: 200 × 200; number of averages: 1; in plane resolution: 100 μm; acquisition time: 10 min 48 s. The T1-w parameters were as follows: 3D Fast Low Angle Shot (FLASH) sequence: TE: 1.9 ms; TR: 5 ms; flip angle: 60°; acquisition matrix: 128 × 128; number of averages: 4; in-plane resolution: 156 μm; acquisition time: 5 min 28sec. To assess iron oxide NP uptake we used a customized T2*-weighted gradient echo sequence 25 and acquired pre- and 48 hours post-contrast scans (3D FLASH sequence, 80 µm isotropic resolution, TE: 18 ms; TR: 50 ms; flip angle: 12°; number of averages: 1, acquisition matrix: 400 x 400, acquisition time: 15 min 40 sec). Pre-contrast images were used to differentiate susceptibility artifacts caused *e.g.* by tumor microbleedings from vessel signals that were only detectable after contrast administration. For MR imaging, anesthesia was induced with 3% isoflurane and maintained with 1-2 %. Animals were kept on a heating pad to keep the body temperature constant. Animal respiration was monitored externally during imaging with a breathing surface pad controlled by an in house developed LabView program (National Instruments Corporation, Austin, TX, USA). Eye cream (panthenol 5%) to avoid eye drying was applied during every anesthesia.

### Iron oxide nanoparticles

Dextran cross-linked iron oxide nanoparticles (CLIO-FITC or CLIO-TAMRA) were synthesized as previously described 26 and had the following specifications: particle size: 32 nm; zeta potential: 6.62; R1: 27.34 mM/s; R2: 72.17 mM/s; 15.5 fluorescent molecules per particle (kind gift of R. Weissleder, MGH, Boston, MA, USA). Animals were injected intravenously by tail vein injection at a dose of 15 mg/kg. MR-MPM was performed 10-14 days after tumor inoculation. MR-MPM scans were performed before, directly after intravenous injection (blood pool phase) and 48h after NP administration to allow phagocyte loading (parenchymal phase). For the quantification of MRI NP uptake, T2* hypointense areas within the tumor (intratumoral susceptibility signals) were segmented semi-automatically before and 48 hours after NP administration (n=3 mice) using AMIRA (FEI, Hillsboro, Oregon, USA). To assess NP-uptake under cell culture conditions and its effect on cellular polarization, CLIO-TAMRA (100µg of iron / ml) was added to bone marrow derived mouse macrophages cultures as previously described 14. To assess the effect on macrophage polarization, macrophages were prepolarized to an “M1-like” phenotype by 100 ng/mL LPS (Sigma) + 10 ng/mL IFNγ (PeproTech) or M2-macrophages by 10 ng/mL IL4 (PeproTech) + 10 ng/mL IL13 (PeproTech). After polarization, CLIO-TAMRA was added for 24 hours. Cells were washed twice with PBS and imaged at indicated time points by confocal microscopy or MRI using a dedicated coil 14. To assess the polarization of macrophages after NP incubation, CD45^+^/CD11b^+^ were stained with MHCII (M1 marker, BioLegend) or arginase (M2 marker, BioLegend). Marker expression was quantified by flow cytometry on a FACSCanto II (BD Biosciences) and analyzed by FlowJo X (BD Biosciences).

### Multiphoton microscopy

MPLSM imaging was performed with a Zeiss 7MP microscope (Zeiss, Jena, Germany) equipped with a Coherent Chameleon UltraII laser (Coherent, Santa Clara, CA, USA) and images were acquired by the ZEISS ZEN Software. A custom-made microscopy stage contained an integrated stereotactic mouse head holder to prevent mouse movement and breathing artifacts and to allow for repeated positioning. For excitation the laser was tuned to 850nm. An appropriate filter set was used to detect GFP and CLIO-TAMRA: band pass filters were 500-550 nm (to detect GFP) and 575-610 nm (to detect CLIO-TAMRA). Laser power was tuned as low as possible to avoid image saturation, fluorophore bleaching and phototoxicity and depended on the imaging depth, the fluorescence intensity of the fluorophore and the window quality. A z-interval of 3 μm was used. The body temperature of mice was kept constant using a heating pad. Isoflurane concentration was 3% for anesthesia induction and 0.5-1.5% for maintenance.

### Immunohistochemistry

For histological correlation analysis, mice were sacrificed in deep anesthesia by intracardial perfusion with PBS, followed by 4% paraformaldehyde (PFA, Roti-Histofix; Carl Roth, Karlsruhe, Germany). Brains were dissected and processed for standard paraffin histology. Five to ten µm cryostat or microtome sections were cut. Stainings were performed with ionized calcium-binding adaptor molecule 1 (Iba-1) antibody (WAKO, Neuss, Germany) for macrophages/ microglia, endothelical cells (CD31, R&D Systems, Wiesbaden, Germany), CD206 (PE-conjugated, BioLegend, San Diego, CA, USA), CD80 (APC-conjugated, BioLegend) and T cells (CD3, Dako Omnis, Santa Clara, CA, USA) using standard immunohistochemistry protocols. Tile scans (20x of the entire tumor bearing hemisphere) and higher-magnification images (63x) were acquired by confocal microscopy (Zeiss LSM700 or Zeiss LSM710). For the quantification of immunohistochemical stainings 3-5 representative regions from the tumor core, tumor border and healthy cortex were selected. Quantification was performed semi-automatically using a custom-written macro in FIJI 27. For segmentation of Iba^+^-1 cells, the corresponding channel was blurred by a Gaussian filter (sigma=5) and images were manually thresholded depending on signal to noise. A mask of the maxima of the counted particles was created (particle size 20-infinity). Subsequently, segmented cells were counted automatically with the plugin “analyze particles”. For the quantification of NP uptake, the cell mask created above, was applied to the NP channel and mean fluorescence intensity per cell was quantified. For quantifying CD80^+^, CD206^+^ and CD80^+^/CD206^+^ cells, the same macro was used. For quantitative microscopy experiments, all acquisition parameters (*i.a.* laser power and PMT settings) were kept constant.

### Statistical analysis

Data is shown as mean ± S.E.M. Statistical analyses were performed in PRISM Version 7.0 (GraphPad). Two-tailed student's t-tests were used to compare 2 groups. Mann-Whitney test or ANOVA were used to compare multiple groups. p values < 0.05 were considered significant. *denotes p < 0.05; **p < 0.01; ***p < 0.001.

## Results

### Establishing MRI compatible cranial windows

**Figure 1:**
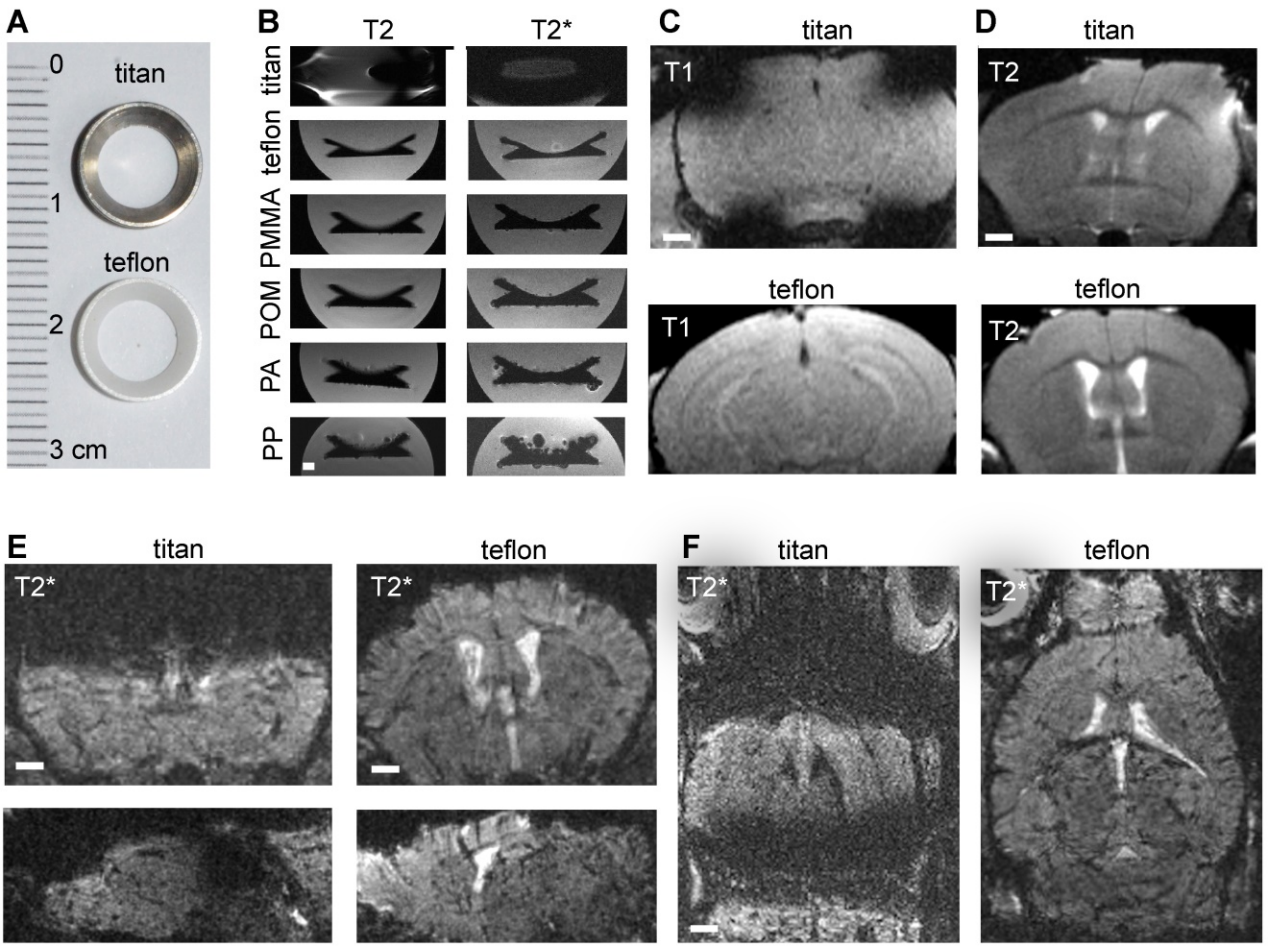
** Comparing MRI properties of Titanium and Teflon cranial windows.** Photograph of Titan and Teflon rings used for cranial windows (a). T2 and T2* MRI images of cranial rings made from Titanium, Teflon polymethylmethacrylate (PMMA), polyacetale (POM), polyamide (PA) and polypropylene (PP) (b). Rings were measured in falcon tubes embedded in agarose. Representative MR images of healthy control animals implanted with Titan or Teflon cranial windows for multiphoton microscopy. T1-w and T2-w images (c,d) are shown. Teflon rings reduce metal artifacts compared to conventional titanium rings. Coronal, sagittal and axial plane T2*-w images are shown (e,f). Scale bar is 1mm.

In order to image innate immune cell dynamics in the tumor microenvironment of glioma bearing mice by MRI and correlated multiphoton microscopy we introduced Teflon cranial windows as holding rings for multiphoton microscopy (Figure 1a). First, we systematically compared cranial rings made from different materials regarding MR imaging characteristics. Whereas rings made from Teflon, polymethylmethacrylate (PMMA), polyacetale (POM), polyamide (PA) and polypropylene (PP) did not cause susceptibility artifacts, titan rings resulted in permissive field distortions (Figure 1b-d). As POM, PA and PP were deformable, we further investigated Teflon rings. In comparison to the conventional Titanium ring that is commonly used for cranial windows, Teflon rings significantly reduced susceptibility artifacts on T1-w and T2-w sequences. The improved image quality was especially apparent on T2*-w images (Figure. 1e-f). This is important for our experimental approach because cortical glioma areas that are amenable for multiphoton microscopy (MPM imaging depth penetration is ~500µm to 1mm) are otherwise completely covered by susceptibility artifacts. Also, the T2*-w sequence used for NP imaging is highly sensitive for susceptibility artifacts (**Figure 1e-f**).

### MRI and multiphoton microscopy for glioma imaging in the same animal

**Figure 2:**
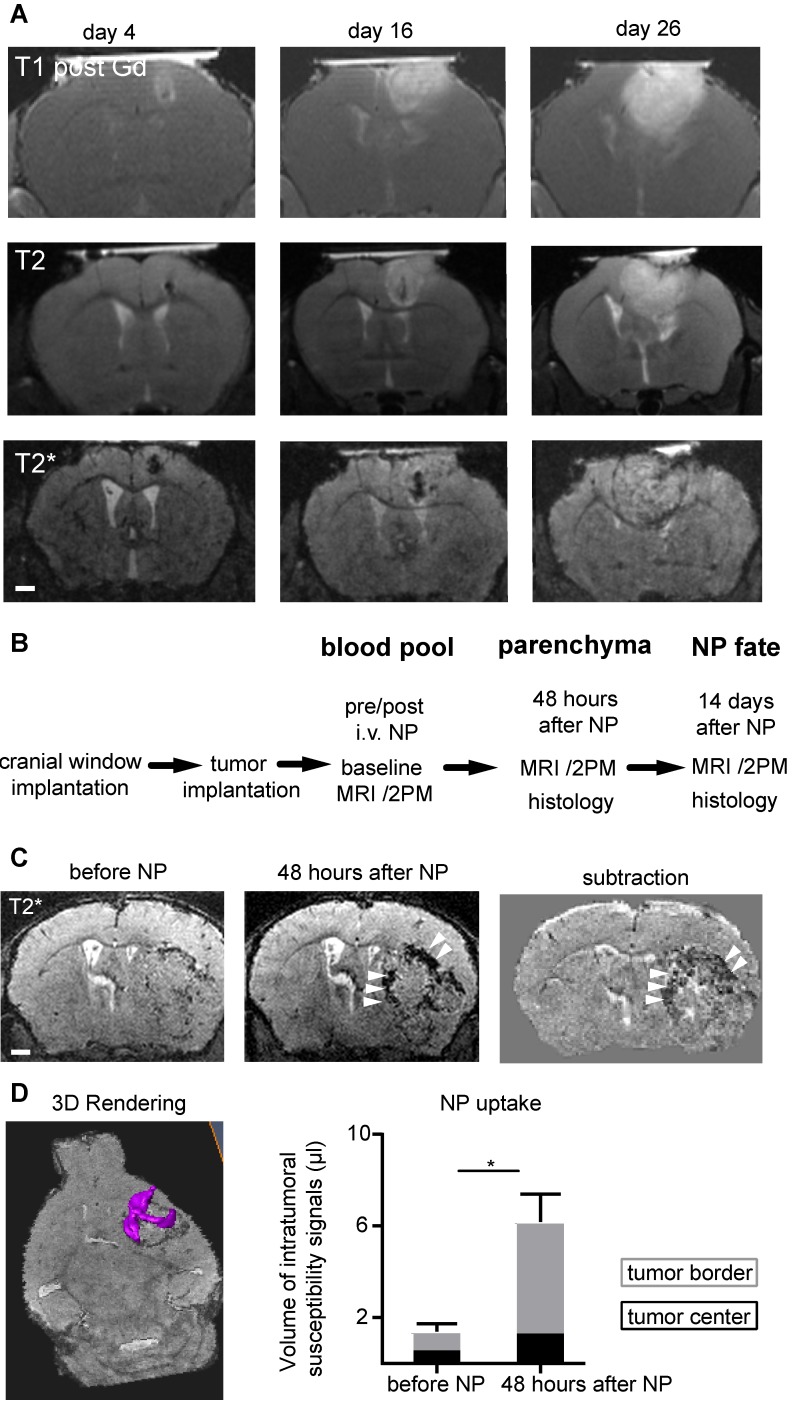
**MRI compatible cranial windows for glioma monitoring.** T1-w imaging after Gd-contrast administration (upper row), T2-w images (middle row) and T2*-w images (bottom row) at day 4, 16 and 26 after tumor inoculation show the rapid tumor growth (a). Study outline shows the experimental and imaging timepoints (b). T2*-w images before and 48 hours after iron oxide nanoparticle (NP) administration (c). Segmentation and quantification of intratumoral susceptibility signals (ITSS; magenta) before and 48 hours after NP administration (d). n=3 mice. ITSS were quantified separately for the tumor border (grey) and tumor core (black bar). For the subtraction image in (c) the T2* image before NP injection is subtracted from the 48 hours post NP image. This facilitates the detection of the hypointensities mainly present in the tumor border (arrowhead) and the differentiation of preexisting tumor microbleedings from NP uptake. Scale bars are 1mm.

The improved image quality with Teflon rings allowed longitudinal imaging of tumor growth kinetics by MRI and multiphoton microscopy in the same animal. Gl261 tumors grew exponentially with increasing tumor mass effect over time (Figure 2a) (20). To visualize innate immune cell dynamics in the TME we used a previously established iron oxide nanoparticle platform 14 in Cx3cr1^+/-^-GFP mice that have fluorescently-labeled myeloid cells, including macrophages/microglia 21. We assessed different phases of NP kinetics starting 14 days after tumor implantation (Figure 2b). Before NP injection T2*-w MRI showed focal spots of hypointensities, most likely tumor microbleedings. Directly after intravenous NP injection, NP were apparent in the circulation outlining vessels. NP were subsequently cleared from the circulation and taken up into the tumor. Focal hypointensities were especially present at the tumor border interface to the healthy parenchyma 48 hours after NP injection (Volume of intratumoral hypointensities before NP: 1.4 µl *vs* 6.1 µl 48 hours after NP administration, p<0.02; Figure 2c-d).

### Iron oxide NP specifically label tumor-associated macrophages and microglia

**Figure 3:**
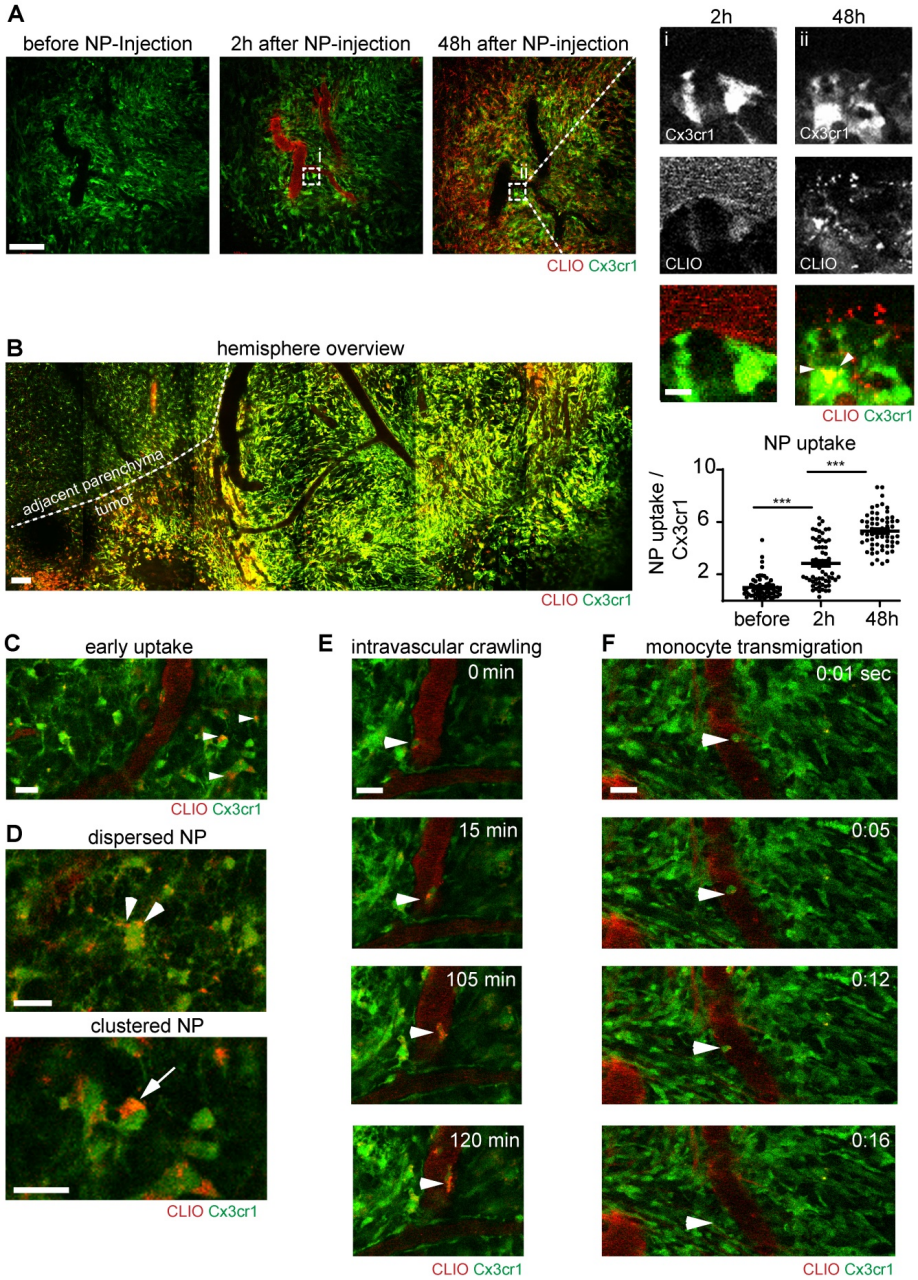
**Immune cell dynamics after nanoparticle administration.** Multiphoton microscopy images of the same region before, 2 hours after and 48 hours after intravenous CLIO-TAMRA injection in a Cx3cr1-GFP animal (a). Close up with single channels show single Cx3cr1^+^ TAMs before and after NP uptake (arrowheads). Quantification of nanoparticle uptake based on fluorescence intensity in TAMRA channel per Cx3cr1^+^ TAM, normalized to baseline (before NP injection). n=60 cells from 3 mice. Overview image 48 hours after NP injection (recorded as tile scan) (b). Dotted line indicates the tumor border. Uptake of NP in brain resident macrophages/microglia two hours after NP injection (c) Different cytoplasmic appearances of NP in TAMs as dispersed (arrowheads) or clustered NP (arrow) (d). Intravascular migration of a monocyte with phagocytosed NP (e). Monocyte transmigration into the adjacent parenchyma (f). Scale bars are 100µm in a, b and 20µm in c-f and magnified images in a. f.i.: fluorescence intensity. a.u.: arbitrary units.

Correlated multiphoton microscopy was performed longitudinally prior to NP injection, directly after intravenous NP injection and 48 hours after injection. The first imaging timepoint after NP administration showed that NP exhibited strong intravascular fluorescent signal and remained mainly intravascularly (Figure 3a). 48 hours after NP injection, there was prominent labeling of a large fraction of the macrophage/microglia compartment (Figure 3a, Figure S1a). The density of macrophages/microglia (Cx3cr1^+^-cells) was strongly increased in the tumor bearing hemisphere (Figure 3b). Immediately after NP injection small amounts of NP also “leaked” into the affected brain parenchyma and got phagocytosed by resident microglia (Figure 3c). Interestingly, there were different stages of NP uptake: we found sparse, punctuated NP uptake in cytoplasmic processes in some macrophages/microglia, whereas other macrophages/microglia exhibited large cytoplasmic NP clusters (Figure 3d). Additionally, there was also immediate labeling of blood-circulating monocytes after NP injection, some of which adhered to the endothelium and transmigrated to the TME to become tumor-associated macrophages (Figure. 3e,f, movie S1,2). Although, there was clear, linear NP association at the vessel wall (Figure S1a/ iii), immunohistochemical staining for CD31 showed no direct co-localization with NP indicating a paracellular uptake mechanism into the brain parenchyma (Figure S1b). Cx3cr1^+^ cells also showed dynamic cytoplasmic protrusions 28 that seemed to actively engulf free NP (Figure S1c, movie S3). In additional *in vitro* experiments we used bone marrow derived macrophage cultures to further dissect the NP uptake kinetics: we found that macrophages accumulate NP over time with increasing cytoplasmic NP uptake (Figure S2a,b). Uptake occurred in a time dependent manner and got saturated 24 hours after NP application (Figure S2b).

### Nanoparticle uptake is specific for TAMs and NP are mainly taken up in the tumor border in M1-like macrophages

**Figure 4:**
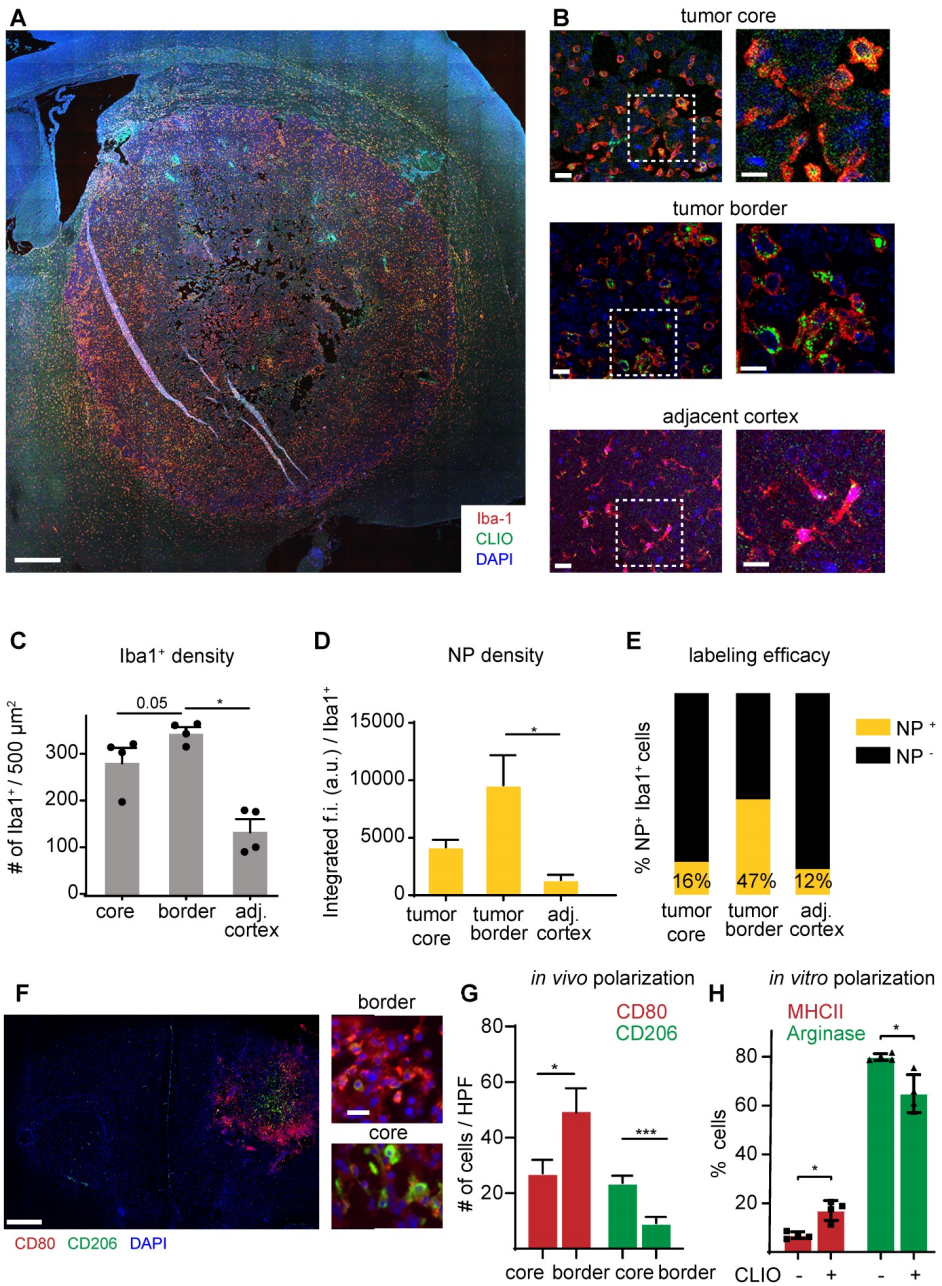
**Immunohistochemistry of TAMs and quantification of NP uptake.** Overview image of the tumor bearing hemisphere (a). Close up and magnified images of the tumor core, periphery and adjacent healthy cortex (b). Quantification of Iba-1^+^ cells (c), the NP uptake (d) and proportion of NP-labeled cells (e) in the different regions. n=4 mice. Immunohistochemical staining of the M1 marker CD80 and M2 marker CD206 (f). Quantification of M1-like and M2-like macrophages / microglia in the tumor core *vs* tumor border. n=6 mice (g). FACS quantification of macrophage polarization as assessed by MHCII and arginase expression. Macrophages were incubated for 24 hours with 100µg of NP and pre-polarized with Il-4/ Il-13 (h). f.i.= fluorescent intensity. Scale bars are 500µm in a, f, 50µm in b and 10µm in close-ups.

To further investigate the specificity of NP imaging we performed *in vitro* and *in vivo* analysis of NP uptake by other cell types of the TME: cultured Gl261 tumor cells showed avid uptake of iron oxide NP *in vitro* (Figure S3a). However, in contrast to the strong *in vitro* uptake, we did not find detectable NP uptake of Gl261-GFP tumor cells *in vivo* (Figure S3b). Also, T cells, although found in high amounts within the TME, did not show detectable NP levels (Figure S3c). Rather, immunofluorescence stainings showed that NP accumulation was restricted to TAM infiltrates that were particularly pronounced at the tumor border (Figure 4a,b). Quantitative assessment of macrophages / microglia showed highest TAM numbers in the tumor border compared to the tumor core and healthy cortical regions adjacent to the tumor (345±22 Iba^+^ cells / field of view in the tumor border *vs* 136±48 in the healthy cortex, p=0.02; n=4 mice Figure 4c). TAMs in the tumor border also showed the highest proportion of NP^+^ cells and engulfed NP to the highest extent (Figure 4c,d). When assessing the TAM phenotype with M1 (CD80^+^) and M2-markers (CD206^+^), the distribution of both populations was markedly different with M1-like macrophages mostly populating the tumor border, whereas M2-like macrophages were mostly present in the tumor core. (M1/M2 ratio in the tumor border: 7.4±3.8 *vs* 1.6±1.3 in the tumor core, p<0.01; Figure 4f-g). Interestingly, NP administration on M2-polarized macrophages led to an increase of MHCII expression and down-regulation of arginase, indicating that NP-uptake by itself leads to a pro-inflammatory shift of macrophages (Figure 4h).

## Discussion

MRI is routinely used for primary diagnosis and clinical follow-up of glioma patients. Multiphoton microscopy and cranial windows are heavily used in basic neuroscience research to investigate physiological and pathological mechanisms on the molecular and cellular level 29, and increasingly also for unprecedented insights into brain tumor biology and their response to therapies 15, 30-32. These two domains have so far been separated because conventional Titanium cranial windows that are used in 2-photon microscopy were incompatible with MRI due to severe metal artifacts and the distortion of the magnetic field caused by the Titanium ring. We have resolved this issue by using Teflon cranial rings that do not disturb the magnetic field and thus allow the acquisition of artifact-free MR images. We used this approach to map the innate immune cell compartment of the glioma tumor microenvironment using fluorescently labeled iron oxide nanoparticles and transgenic macrophage / microglia reporter mice (Cx3cr1-GFP). These NP are avidly phagocytosed both by circulating monocytes that are recruited to the TME as well as by brain resident microglia. These bimodal nanoparticles result in a hypointense signal drop on MR images and are additionally detectable by *in vivo* microscopy via their fluorescent tag. In line with our previous study in a neuroinflammatory model of multiple sclerosis (MS) 14, we found that NP are primarily taken up by circulating monocytes, tumor macrophages and brain resident microglia whereas tumor cells and T cells phagocytosed NP only sparsely. We further validated our MR imaging findings by immunofluorescence microscopy. Here, we found that the MR signal mainly originates from macrophages / microglia that accumulate in the tumor border where they also showed the highest uptake of NP. Interestingly, the tumor border was enriched in pro-inflammatory, M1-like macrophages whereas M2-like macrophages were mainly found in the tumor core, indicating a spatial separation of the two macrophage phenotypes. Few macrophages also expressed both markers, possibly indicating a transdifferentiation capacity of the TAM compartment, similar to recent findings in an MS model 33. Previous studies have suggested that NP uptake by itself leads to an induction of M1-like macrophages which results in an anti-tumor effect 34, 35. This concept was recently further substantiated with a myeloid targeting nanoparticle that acted synergistically with checkpoint inhibition by inducing an M1-shift in TAMs 36. NP imaging has also recently been taken to clinical glioma imaging, where NP imaging could be used as an imaging biomarker to quantify TAMs in the glioma TME 37.

Conceptually, we found various routes of NP into the brain: First, blood-circulating monocytes take up NP immediately after intravenous injection. Some of these labeled monocytes are then recruited as tumor-associated macrophages to the TME. Secondly, NP can leak directly into the brain parenchyma in areas of blood brain barrier disruption where they are taken up by brain resident microglia. Both effects result in a comprehensive labeling density of the innate immune cell compartment within the tumor of ~50% (**Figure 4e**). Given such a high accessibility of TAMs by NP we envision NP imaging as one possibility to assess and possibly modulate the TME using theranostic NP as a carrier for either pharmacological or genetic therapeutic interrogation 38 that might act synergistically to immunotherapies 39. Furthermore, the TME can be massively modified by immunotherapeutic approaches that are currently developed in a preclinical setting, are tested in clinical trials and start to enter clinical practice 10, 40-42, all of which need monitoring by imaging. NP imaging could constitute one possible approach.

Limitations of our study include the different scales of our imaging approaches that made direct co-registration of MRI and multiphoton microscopy difficult. We rather compared different regions of the tumor (border *vs* core) instead of direct image co-registration. Additionally, quantification of iron oxide nanoparticles on MR images and the determination of exact cell numbers by MRI were not possible in our study. NP act as a negative contrast agent and lead to a sustained signal drop on T2*-w images. This signal decrease is also caused by other sources of susceptibility, *e.g.* deoxygenized blood within (tumor) microbleedings. That is why careful pre and post MR imaging is necessary to delineate susceptibility signals that are newly occurring after NP administration and thus can be attributed to TAMs. In macrophage cultures, we found that fluorescence intensity per cell decreases slowly over time, suggesting that the NP gets slowly degraded upon uptake. However, both, fluorescence intensity and iron were still detectable 7 days after administration *in vitro* indicating slow metabolic breakdown of the particle (Figure S4a-e). This was mirrored by long tissue half-lives *in vivo*: when assessing the long-term fate of NP in glioma bearing mice we found that two weeks afterNP injection there was still detectable NP within macrophages / microglia in the TME (Figure S4f). It is generally assumed that the NP iron gets released over time and replenishes the body iron reservoir. Actually, the clinically approved iron oxide formulation ferumoxytol is used for treating iron deficiency anemia and only utilized “off label” as a contrast agent. Ferumoxytol has proven as a safe agent and has been used in numerous clinical trials 43-46. Nevertheless additional studies of biodistribution and clearance of NP seem mandatory before taking NP imaging to the clinical arena, especially for neuro-applications with a compromised blood-brain barrier, also given the current safety concerns of Gd-contrast agent retention in the brain 47.

In summary, our study establishes MR-MPM with iron oxide NP as a technical platform for multi-modality imaging of innate immune cell responses in the glioma tumor microenvironment. We envision that NP imaging along with similar approaches 48 will be important to establish imaging biomarkers for immunotherapeutic interventions and the alterations that occur on the cellular level during therapy which cannot be directly assessed by current clinical imaging.
