# Supplementary material for: Monitoring innate immune cell dynamics in the glioma microenvironment by magnetic resonance imaging and multiphoton microscopy (MR-MPM)
Source: Theranostics. 2020 Jan 1;10(4):1873–83. doi: 10.7150/thno.38659 (PMC6993231; doi:10.7150/thno.38659)
Supplement: Supplementary file 1 — Supplementary figures. [file thnov10p1873s1.pdf]

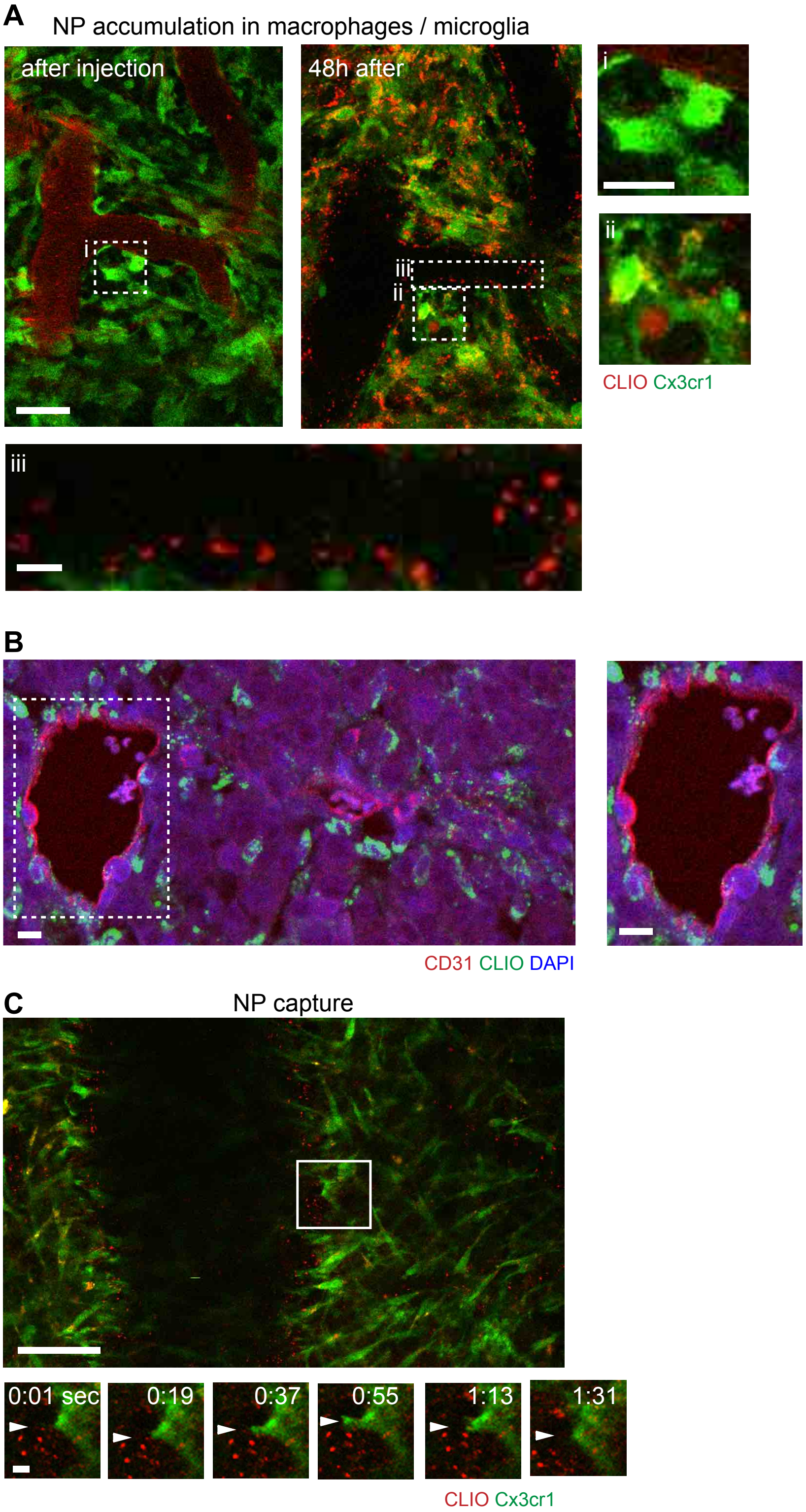

SFig. 1, Karimian et al, Correlated MRI-2PM

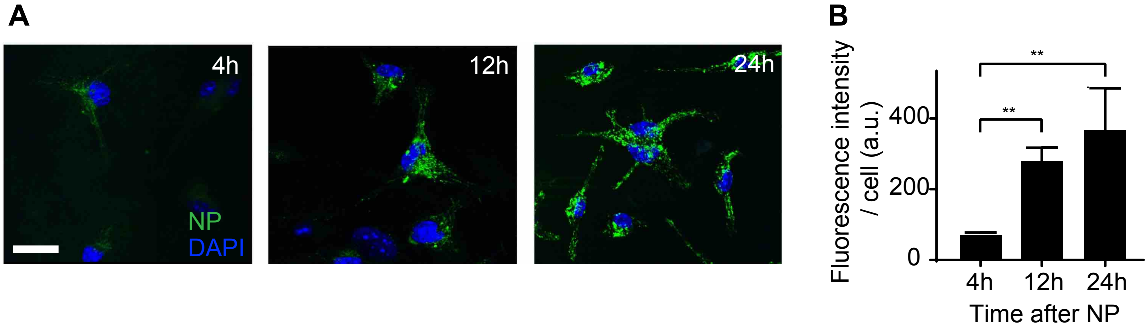

Suppl. Fig. 2, Karimian et al, Correlated MRI-2PM

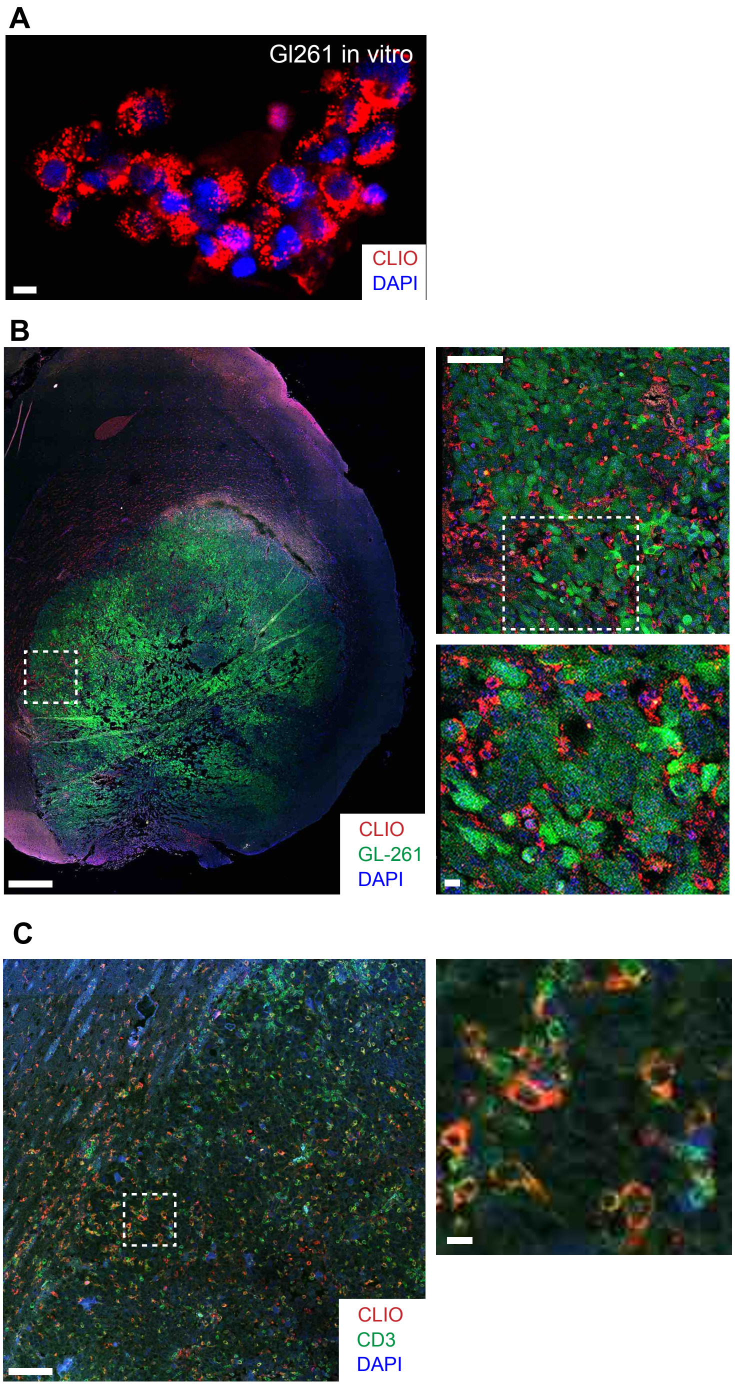

SFig. 3, Karimian et al, Correlated MRI-2PM

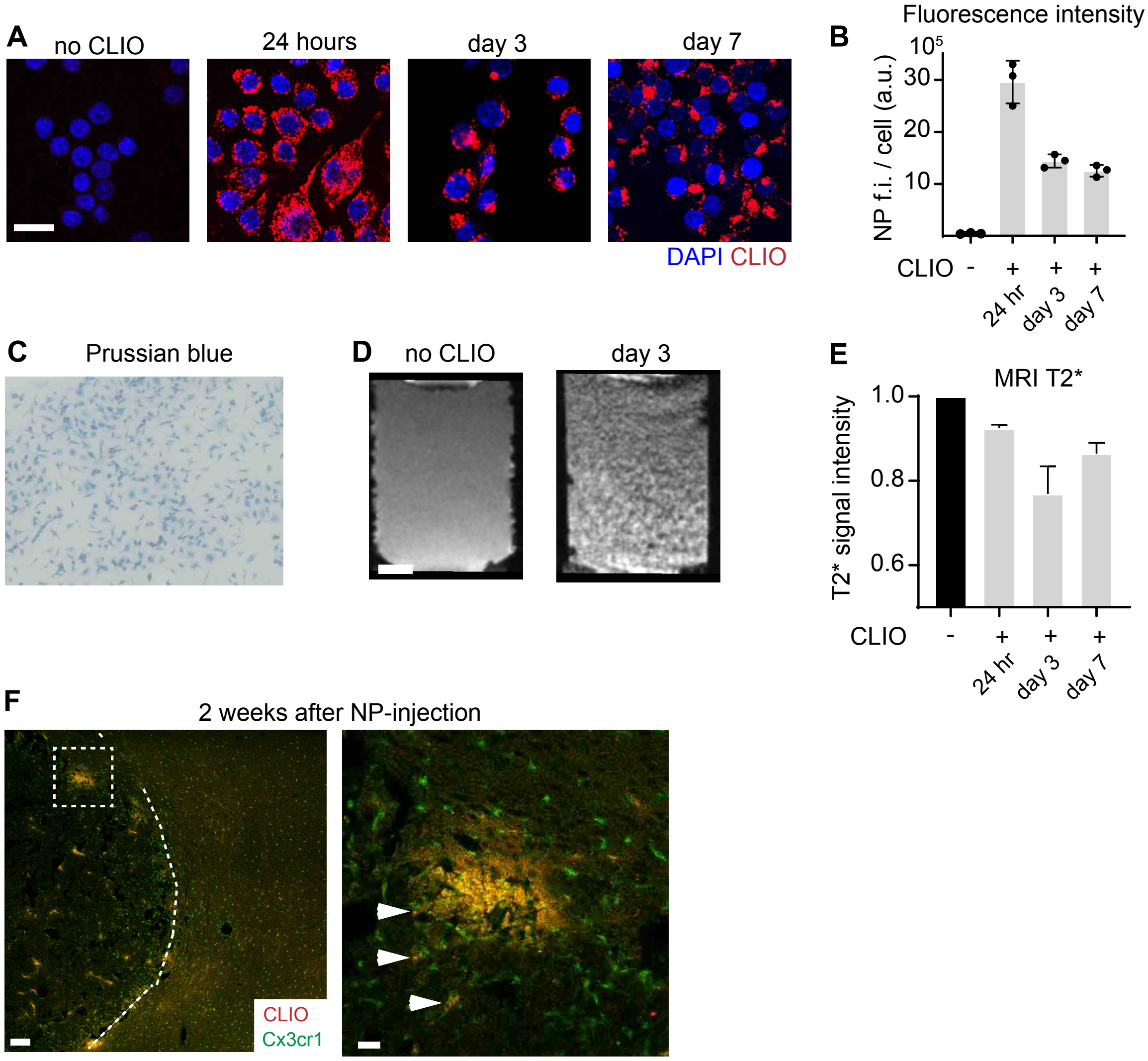

SFig. 4, Karimian et al, Correlated MRI-2PM
